# Impoverishment, hunger, and pandemic: Emergency Aid, the end of the Bolsa Família Benefit, and its replacement, Auxílio Brasil, 2019-2022

**DOI:** 10.1590/S0104-59702023000100032

**Published:** 2023-08-14

**Authors:** Denise De Sordi

**Affiliations:** i Pesquisadora de pós-doutorado, Casa de Oswaldo Cruz/Fiocruz. Rio de Janeiro – RJ – Brasil denisends@me.com

**Keywords:** Bolsa Família, Social welfare system, Minimum wage, Auxílio Brasil, Covid-19, Programa Bolsa Família, Sistema Único de Assistência Social, Renda mínima, Auxílio Brasil, Covid-19

## Abstract

This article addresses the political choices made by the Brazilian government concerning conditional cash transfer programs during the covid-19 pandemic. The aim is to analyze how the covid-related Emergency Aid (Auxílio Emergencial), the extinction of the Bolsa Família Program, and the implementation of its replacement, Auxílio Brasil, interacted in the rearrangement and dismantling of Brazil’s social protection network in a broader context of a generalized impoverishment of Brazilian workers. The article not only presents a record of these actions, but also offers an interpretative approach that, historically contextualized, can shed light on these actions, which ran parallel to the deepening entrenchment of neoliberal policies in the country.

## Resumo

O artigo aborda as escolhas políticas do governo brasileiro relacionadas aos programas sociais de transferência condicionada de renda feitas em meio à pandemia de covid-19. O objetivo é analisar como a implementação do Auxílio Emergencial, o fim do Programa Bolsa Família e a implementação do Auxílio Brasil estão relacionados ao rearranjo e ao desmanche da rede de proteção social brasileira diante da conjuntura de generalizado empobrecimento dos trabalhadores brasileiros. Espera-se não só apresentar um registro dessas ações, mas estabelecer chaves de leitura que, contextualizadas historicamente, permitam explicar a análise de tais ações em meio ao aprofundamento das políticas neoliberais no país.

Em agosto de 2021, considerado o ano mais letal da pandemia no Brasil, com 619.109 pessoas mortas por covid-19, e no qual a degradação das condições materiais de vida dos trabalhadores se agravou em ritmo não observado anteriormente (Campello, Brandão, 2 jun. 2022; Amorim, Neder, 10 jun. 2022) com o retorno do país ao Mapa da Fome (Pajolla, 30 jun. 2021; Penssan, 2021, 2022), o Programa Bolsa Família (PBF), exemplo mundial de programa de transferência condicionada de renda, foi encerrado e, em seu lugar, foi implementado o Programa Auxílio Brasil (PAB). Esse ato demarcou uma virada conservadora na coesão social em torno dos programas de transferência condicionada de renda e dos instrumentos de gestão da pobreza pelo Estado. Se o PBF era um programa de destaque por vincular uma gama de serviços sociais voltados para a redução das desigualdades sociais, seu conteúdo social foi progressivamente esvaziado e, ao longo da pandemia, com a aprovação do Auxílio Emergencial, foi abandonado enquanto instrumento principal para o combate ao aumento da pobreza e da fome no país (De Sordi, 24 ago. 2021, 24 fev. 2021).

Desde o início do governo de Jair Bolsonaro (Partido Liberal, PL), em 2019, mecanismos de minoração das desigualdades sociais construídos ao longo das décadas de 1990 e 2000 foram sistematicamente paralisados ou tornados inoperantes (De Sordi, 2 fev. 2021). Esse é um tipo de escolha política, muitas vezes justificada pelo governo com o argumento de controle da crise macroeconômica (Audi, 1 nov. 2021) supostamente decorrente das medidas de proteção à vida e à saúde durante a pandemia. Porém, o retrocesso generalizado (Paiva, Teixeira, Pires-Alves, jun. 2020) da rede de proteção social brasileira não pode ser naturalizado, pois atende a um projeto de governo eleito sob a premissa de enxugamento do "gasto social", que encontrou, na desmoralização dos programas e políticas sociais existentes, uma via de concretização.

Nesse ínterim, a hipótese abordada neste artigo, portanto, é de que ocorreu um processo social de ressignificação da condição de pobreza para que fosse possível levar adiante o desmanche da rede de proteção social, provocando a financeirização (Lavinas, Gentil, 2018; Oliveira, 1988) acelerada do acesso aos serviços sociais básicos, antes mediado por programas sociais, a exemplo do PBF.

O argumento é a condição de pobreza ter sido politicamente ressignificada durante o cenário mais agudo da pandemia, em um processo que associou a desmoralização dos instrumentos de gestão da pobreza pelo Estado, taxados como ineficientes, permitindo, com o Auxílio Emergencial, a ampliação do público anteriormente atendido pelos programas sociais de transferência condicionada de renda, de modo a cobrir os efeitos das políticas de desmanche dos direitos sociais sistematicamente iniciadas em 2016, com o *impeachment* que depôs Dilma V. Rousseff (Partido dos Trabalhadores, PT) e alçou Michel Temer (Movimento Democrático Brasileiro, MDB) à presidência.

Tais efeitos do desmanche e da recomposição do Estado brasileiro para atender a uma agenda de reformas macroeconômicas foram agudizados em meio ao cenário de crise política, econômica e social, por sua vez, acentuado pelos efeitos sociais derivados da inação como forma de gestão dos impactos da pandemia pelo governo federal (Balanço indica..., 31 dez. 2021; Arias, 25 nov. 2021; Senador pede..., 12 jan. 2022; Cueto, Neelakantan, Lopes, 2022). Ao ser relida pelo projeto de desmanche da rede de proteção social, a condição de pobreza passou a ser relacionada aos efeitos da gestão da pandemia, descolada de questões estruturais e da responsabilidade do Estado como regulador das condições de reprodução social. O agravamento dessa condição, com a generalização da fome pelo país, por sua vez, é um processo que relê a pobreza, que passa de termo multifacetado (Sen, 2010) para um retorno moral e material no qual “volta a ser sinônimo de fome” (De Sordi, 2019, p.31).

A condição material de pobreza é tomada neste artigo como chave de leitura que permite analisar formas específicas de mediação das relações entre Estado e sociedade. O termo indica, portanto, uma condição material de vida específica que é reproduzida pelo próprio Estado. É certo que falar em “pobreza” significa remeter a uma forma concreta de organização das condições materiais de reprodução dos trabalhadores e de uma condição material de vida dura para quem a enfrenta. Assim, estabelecer essa chave de leitura não é mero exercício de abstração, mas uma forma de análise para a compreensão das bases que foram movidas para que o governo federal pudesse se utilizar do contexto da pandemia para promover, primeiramente o Auxílio Emergencial, implementado em paralelo e a despeito da existência do PBF, e o subsequente encerramento deste último programa para a implementação do Auxílio Brasil, aproveitando-se da popularidade do Auxílio Emergencial (De Sordi, 24 fev. 2021).

A mudança de concepção, formato e execução nos programas de transferência condicionada de renda não está limitada a mera questão técnica, mas caracteriza o aspecto gerencial desses programas (Behring, 2003; Georges, Rizek, Ceballos, 2014), voltados para a minoração – e não para a erradicação – da condição de pobreza, pois são parte de uma agenda de políticas neoliberais (Mauriel, 2008). Entretanto, ainda que em meio a essa agenda, tais mudanças marcam a erosão do horizonte de possibilidades (Vargas-Maia, De Sordi, 2022) e expectativas conquistado pelos trabalhadores no período posterior à redemocratização do país. Horizonte que antes se encontrava sustentado por uma rede de políticas sociais integradas pelos instrumentos de gestão centralizados no Sistema Único de Assistência Social (Suas).

Ao localizar as ações institucionais e as escolhas políticas que resultaram no desmanche dos programas sociais em meio à crise sanitária e econômica, basicamente, este artigo objetiva colaborar para as discussões que tencionam responder – a partir desta dimensão de observação social que são as políticas e programas sociais brasileiros – à pergunta de como foi possível, em tão pouco tempo e após um amplo processo de conciliação social, atingirmos patamares de empobrecimento não observados anteriormente.

Não é objetivo exaurir o debate apresentado, já que sua própria temporalidade impõe dificuldades de avaliação dos aspectos que serão mais importantes na definição do processo histórico (Hobsbawm, 1998) de reformulação das políticas sociais, que é aqui interpretado como uma janela de observação para mudanças no campo mais amplo de organização de nosso tecido social e da própria configuração da democracia no país. O mapeamento apresentado nas seções que seguem é feito de forma orientada por pesquisas anteriores (De Sordi, 2019; De Sordi, Gonçalves-Neto, 2021) que permitiram um olhar temporalmente alargado sobre as permanências e mudanças no processo de reformas dos programas sociais da Nova República. Portanto, a intenção é ler a conjuntura para demarcar historicamente as mudanças que ocorreram, o que elas nos informam e o que permitem explicar sobre a configuração da produção e da reprodução da pobreza no Brasil contemporâneo.

Desse modo, a primeira seção tem por objetivo explicar como o fim do PBF demarca um processo ampliado de erosão do Suas, pilar da rede de proteção social brasileira, e a desestabilização dos instrumentos de controle social que haviam garantido a saída do país do Mapa da Fome em 2014; para tanto, retoma-se brevemente o processo histórico de construção desses instrumentos. Na segunda seção, demarcam-se as diferenças entre o PBF e o PAB, evidencia-se por que este último não pode ser considerado um substituto do primeiro e o que esse novo programa significa em meio ao cenário de crise sanitária, política, econômica e social; reconstituindo, portanto, os elementos de coesão social que tornaram possível finalizar um dos maiores programas de transferência condicionada de renda do país. A última seção, de considerações finais, evidencia a relação entre os elementos principais desse desmanche e os antagonismos em meio aos quais tem ocorrido o aprofundamento das políticas neoliberais no Brasil.

## A rede de proteção social brasileira e o Programa Bolsa Família

Em 2014, o anúncio da saída do Brasil do Mapa do Fome registrou um momento social incomum na história brasileira. De acordo com a então ministra do extinto Ministério do Desenvolvimento Social e Combate à Fome (MDS), Tereza Campello, a saída do Mapa do Fome só havia sido possível mediante o desenvolvimento de "um conjunto de políticas públicas que garantiram o aumento da renda dos mais pobres e um aumento da oferta de alimentos, que consolidaram a rede de proteção social" do país (Sair do Mapa..., 16 set. 2014).

No relatório "O estado da segurança alimentar e nutricional no Brasil: um retrato multidimensional" (FAO, 2014, p.6), a importância da articulação entre ações e programas sociais é destacada como elemento central do êxito na minoração da fome e da pobreza:

> Os avanços no combate à fome e à pobreza decorrem, na análise apresentada no relatório da FAO, da priorização da agenda de Segurança Alimentar e Nutricional (SAN) a partir de 2003, com destaque ao lançamento da Estratégia Fome Zero, à recriação do Conselho Nacional de Segurança Alimentar e Nutricional (Consea), à institucionalização da política de SAN e à implementação, de forma articulada, de políticas de proteção social e de fomento à produção agrícola.

No conjunto dessas políticas públicas destacadas figura o Programa Bolsa Família (PBF),[1] consolidado como referência política para o combate à fome e à pobreza no país, e como um dos principais modelos de programa de transferência condicionada de renda no mundo. Uma referência política, pois, "se tornou uma aparente unanimidade, sendo defendido pela maioria dos organismos internacionais, por vários segmentos da sociedade e praticamente por todos os candidatos das eleições presidenciais de 2010" (Cotta, Paiva, 2010, p.60).

De acordo com o relatório (FAO, 2014, p.8):

> O programa de transferência condicionada de renda Bolsa Família – lançado em 2003, realiza transferências monetárias preferencialmente em nome da mãe ... sob a condição de que as crianças das famílias permaneçam na escola e visitem periodicamente

> os serviços de saúde locais para vacinação e acompanhamento do crescimento. O investimento no programa triplicou nos últimos dez anos, chegando a quase R$25 bilhões em 2013, o que equivale aproximadamente a um terço dos gastos federais em programas e ações de segurança alimentar e nutricional.

Por seus reais méritos e abrangência, o PBF se tornou uma marca dos governos petistas e o programa social de maior destaque do país. Como registrou o então presidente Luiz Inácio Lula da Silva (Partido dos Trabalhadores, PT), o PBF "tornou-se a iniciativa mais conhecida e notada dentre todas do Fome Zero" (Silva, 2010). O programa foi formulado em 2003, primeiro ano do governo Lula, a partir de uma série de experiências e debates que circularam ao longo da década de 1990 e que colocaram o Fome Zero, elaborado, por sua vez, com base na experiência das Caravanas da Cidadania, no centro das ações do governo (De Sordi, 2019, p.138).

Deve-se frisar que, apesar da imagem autônoma usualmente atribuída ao PBF a partir de sua regulamentação em setembro de 2004, esse programa passou a ser compreendido como uma das ações daquilo que se tornou a "Estratégia" Fome Zero (Silva, Tavares, 2010, p.245). Proposta inicial de programa social para a gestão das políticas sociais no governo Lula, o Fome Zero foi politicamente derrotado (De Sordi, 2019, p.21 e 31) – devido a seu caráter estruturalmente transformador conectado às pautas históricas dos movimentos sociais – e figurou como "estratégia" de governo para a área social, abrigando um leque de programas sociais, que ofereceram suporte estrutural para a redução das desigualdades sociais, incluindo o PBF.[2]

O Fome Zero foi construído ao longo dos anos 1990 a partir da leitura de reivindicações mediadas por um cenário público de articulação da relação entre Estado, movimentos sociais e sociedade civil. Representou o acúmulo de experiências derivadas das disputas pela mediação dessa relação, pautada pela implementação dos direitos sociais então recém-aprovados na Constituição de 1988, que forjou a proposta do programa com um viés fincado em políticas sociais mais amplas que envolviam, por exemplo, a reforma agrária (De Sordi, 2019, p.30-51), mas que foi retirado de cena em favor do PBF.

A pauta da reforma agrária, por sua vez, foi pulverizada (De Sordi, 2019, p.88-110) em programas setoriais, a exemplo do Programa de Aquisição de Alimentos (PAA) – extinto em 2021, junto ao PBF – e em políticas relacionadas à soberania alimentar geridas pelo Conselho Nacional de Segurança Alimentar e Nutricional (Consea). O PAA foi estruturado de modo a operacionalizar

> a compra direta de alimentos dos agricultores familiares pelo governo, que faz doação às instituições que atendem populações vulneráveis, para uso na merenda escolar ou para repor os estoques governamentais. Em 2012, mais de 185.000 agricultores de todo o Brasil participaram do programa, cada um recebendo em média, mais de R$ 4 mil por seus produtos. Os recursos federais para o Programa aumentaram quase dez vezes desde 2003, sendo superiores a R$ 1,3 bilhão em 2013 (FAO, 2014, p.8).

No âmbito desse rol de programas sociais, o Consea cumpriu papel fundamental na orientação e coordenação dos programas ligados à segurança alimentar e no monitoramento da fome no país. A criação do conselho, em 1993, derivou da pressão política exercida pelo campo de oposição aglutinado em torno do processo de *impeachment* de Fernando Collor, do governo de Itamar Franco pela implementação de um plano de Segurança Alimentar e Nutricional (SAN) e da divulgação do Mapa da Fome em 1993 (De Sordi, 2019, p.38-41). Desde sua criação, o conselho foi alvo de disputas políticas centrais no tensionamento entre políticas universais e focalizadas, bem como da concepção de bem-estar que seria incorporado pelo Estado brasileiro. Na primeira Conferência Nacional de Segurança Alimentar, eram apresentadas pautas interligadas ao conceito de segurança alimentar que incluíam: "a relação Estado-sociedade, ações locais de cidadania, a questão agrária e o desenvolvimento rural, política agrícola, saúde, educação, geração de empregos e políticas de renda" (Telles, 2013, p.153).

Não de modo despropositado, o Consea foi desativado em 1995 por Fernando Henrique Cardoso (Partido da Social Democracia Brasileira, PSDB) em favor da instalação do Programa Comunidade Solidária que, em síntese, desarticulou a proposta de gestão comunitária e política dos programas de soberania alimentar em favor de incentivos do governo ao terceiro setor e ações majoritariamente desenvolvidas em nível local. A interdição da gestão social do Consea reaparecerá como pauta na proposta de programa do Fome Zero, por meio da implementação de Comitês Gestores que serão desativados com a reformulação do Cadastro Único para programas sociais do governo federal (CadÚnico), em 2003. A reativação do Consea nesse mesmo ano, por sua vez, junto à proposta do Fome Zero, ocorreu no sentido de viabilizar a centralidade da pauta da fome e da pobreza na agenda política nacional a partir da campanha presidencial de Lula em 2002 (De Sordi, 2019, p.33 e 58). O Consea, entre 2003 e 2019, teve por papel "fazer recomendações e monitorar as políticas de segurança alimentar e nutricional, incluindo o Plano Nacional de Segurança Alimentar e Nutricional", de modo a promover "a integração das ações em uma estratégia unificada" (FAO, 2014, p.7).

Cabe sublinhar que o PBF emergiu em um espaço político de adequação e conciliação de antagonismos sociais (Braga, 2016) no contexto social de início dos anos 2000, no qual o desenvolvimento de programas sociais de transferência condicionada de renda respondeu a um cenário caracterizado pela intensificação da exploração do trabalho, do aumento do número de trabalhadores empobrecidos e da disputa por diferentes formatos de programas nacionais para a assistência social. O objetivo do PBF de minorar a pobreza a partir de ações sociais focalizadas alterou o horizonte de possibilidades para a universalização dos direitos sociais e atendeu às expectativas de reformas macroeconômicas orientadas por uma agenda neoliberal (De Sordi, 2019, p.105-125). Ao longo de sua existência, o PBF foi ampliado, sofreu modificações em seu sistema de monitoramento e passou a incorporar estratégias diversas, tais como Brasil Sem Miséria e Brasil Carinhoso.[3]

Entre as ações que estruturaram não só o PBF, mas a gestão das políticas sociais no país, está o CadÚnico. Criado em 2001, durante o governo de Cardoso, a proposta de sua utilização para todos os programas sociais do governo federal estava contida no lançamento do Cartão Cidadão, que unificou os programas sociais do período. Com o Fome Zero, o Cartão Cidadão foi substituído pelo Cartão Alimentação, que universalizava a renda para os sujeitos empobrecidos e rompia com a premissa da condicionalidade do Bolsa Escola Federal, programa articulador da implementação do Cartão Cidadão. Porém, com a derrota política do Fome Zero e o lançamento do PBF, foi preciso incorporar os instrumentos de gestão já normatizados, e o CadÚnico sofreu uma reformulação em 2003, passando a ser utilizado com o objetivo de "controlar a focalização do programa nos pobres" (Cohn, 2010, p.223) e desmobilizando a proposta de Comitês Gestores, de base popular, que caracterizaria a gestão do Fome Zero.

O CadÚnico, entretanto, se tornou a maior "base informacional" (Cf. Sen, 2010, p.78-83) dos programas sociais no país a partir de 2003; longe de ser apenas uma simples plataforma de cadastro de dados populacionais, tornou-se crucial para o funcionamento dos programas sociais, já que todo programa direcionado à população considerada de baixa renda estava obrigado a utilizá-lo.[4] Em resumo, o CadÚnico, ao ser utilizado para a manutenção da focalização desses programas – em detrimento do preceito da universalização dos direitos sociais e da gestão democrática ampliada dos programas sociais, acabou por permitir o diagnóstico e o acompanhamento sobre o crescimento ou a redução dos números que medem a condição de pobreza no país, direcionando metas de atendimento e mesmo repasses de verbas aos municípios por meio do Índice de Gestão Descentralizada (IGD).[5]

O CadÚnico teve como efeito colateral se tornar uma base de dados para pesquisas e para o desenvolvimento de políticas públicas, contribuindo para a conservação de patamares aceitáveis de reprodução da pobreza, uma vez que abriu campo para a circularidade de dados e pesquisas sobre os programas sociais, definindo, portanto, a agenda concêntrica de investigações sobre a pobreza e o que é, ou não, relevante para os programas, permitindo a continuidade da busca por soluções técnicas (Cf. Berhan, 2013, p.285-286; De Sordi, 2019, p.24-26) para problemas conectados à formação social do país.

Nesse ínterim, cabe sublinhar que a rede de proteção social brasileira – em sua totalidade, composta pela assistência social, saúde e previdência – teve a assistência social formalizada em âmbito nacional a partir da implementação, em 2005, do Sistema Único de Assistência Social, com a porta de entrada para parte dos serviços dessa rede articulada nos Centros de Referência de Assistência Social (Cras), que a partir do atendimento socioassistencial, apoiado no CadÚnico, promovia o acesso da população aos direitos sociais por meio do cadastro em programas sociais. Assim, o processo de erosão dessas políticas, ainda que elas pareçam conectadas somente a aspectos técnicos de gestão, precisa ser observado em conjunto e contextualizado no quadro histórico, político e social que permite evidenciar a dimensão do desmanche recente.

A interligação dessas ações, formas de gestão e concepções do campo socioassistencial é uma das vias de compreensão dos motivos pelos quais as ações de desmonte de programas sociais e de seus instrumentos de gestão, apesar de muitas vezes indicarem ações setorizadas ou ligadas a meras questões técnicas, evidenciam ações de desestabilização da rede de proteção social em seu conjunto, pois, para o objetivo de minoração da pobreza, os programas e instrumentos explicitados não funcionam desarticulados uns dos outros. Analisar o processo de erosão da rede de proteção social brasileira, portanto, demanda situar o desmonte das políticas sociais criadas entre 1990 e 2014, observando suas funções a partir de um quadro maior, isto é, de forma interligada e relacionada para localizar os diversos atos evidenciadores de que este foi um tipo de escolha sistemática do governo brasileiro, acentuado a partir de 2019.

Apesar dos avanços e méritos reais atingidos com a gama de ações socioassistenciais promovidas na dimensão do Suas, e pelo PBF, desde o início do mandato presidencial de Jair Bolsonaro em 2019 os programas e políticas sociais voltados para a minoração da extrema pobreza e da pobreza, antes garantidores da Segurança Alimentar e Nutricional no Brasil, foram alvo da agenda de reformas políticas e econômicas que levou o país a retornar ao Mapa da Fome.

Em fins de 2020, segundo dados da Rede Brasileira de Pesquisa em Soberania e Segurança Alimentar (Penssan, 2021), havia 116,8 milhões de pessoas em condição de insegurança alimentar, sendo 19,1 milhões em situação de fome. Em fins de 2021, de acordo com a Pesquisa Nacional por Amostras de Domicílios Contínua, "com o rendimento médio de R$415 por mês para a metade mais pobre, são 106,35 milhões de pessoas subsistindo com apenas R$13,83 por dia. O empobrecimento é histórico mesmo quando se compara com quase dez anos atrás: em 2012, a metade mais pobre da população ganhava R$448 por mês por pessoa. O valor de 2021 é 7,4% menor do que o registrado em 2012" (Amorim, Neder, 10 jun. 2022). É possível indicar que a finalização do PBF em 2021 significa um ponto de encerramento da estrutura de gestão dos programas sociais no país, pois esta foi elaborada a partir de um processo cumulativo de experiências sociais, de reivindicações populares e da nacionalização das possibilidades de acesso aos direitos sociais, sob o objetivo da minoração da pobreza.

É simbólico que um dos primeiros atos de Bolsonaro ao assumir a presidência do país tenha sido extinguir o Consea em nível nacional,[6] em sinalização favorável ao setor agroindustrial, e de fechamento de diálogo com os movimentos sociais e a sociedade civil organizados em torno da luta por medidas de agricultura familiar, reforma agrária e soberania alimentar. O fechamento do Consea e os atos que seguiram e que evidenciam o processo de erosão da rede de proteção social estão localizados no interior de escolhas sistemáticas do governo brasileiro que ignoraram – em favor da agenda econômica – o acúmulo de experiências do país na área e que haviam promovido a saída do Mapa da Fome em 2014 a partir da concepção de que a "Segurança Alimentar e Nutricional não é um conceito técnico, é um conceito político, construído com intensa participação social" (FAO, 2014, p.16).

Indicar que o retorno do país ao Mapa da Fome a partir de fins de 2020 (Penssan, 2021, 2022) – no contexto da pandemia – é uma escolha deliberada do governo de Bolsonaro não é afirmação retórica. Entre os fatores determinantes para o êxito das políticas de SAN estão a estabilidade política, o atendimento ao custo das necessidades básicas, empregos formais e a existência de uma rede socioassistencial (FAO, 2014, p.18), todos direitos sociais previstos na Constituição de 1988, operacionalizados no âmbito do Suas, porém sistematicamente desativados ou tornados inoperantes ao longo do mandato de Bolsonaro.

## O fim do Programa Bolsa Família e o Auxílio Brasil

Ao assumir o cargo de presidente em 2019, Bolsonaro já havia explicitado sua interpretação acerca dos programas sociais (Mendes, 10 ago. 2021; Bolsonaro..., 28 out. 2021) e o projeto político que levaria a cabo durante seu mandato presidencial, priorizando o "enxugamento do custo social" com uma política econômica comandada pelo *Chicago*

*boy* Paulo Guedes (Anderson, 2019, p.184), com orientação fiel ao teto de gastos aprovado no governo de Michel Temer.

Em 2018, durante a campanha eleitoral à presidência, corriam publicamente declarações da chapa do então candidato Bolsonaro por meio das quais se estabeleceu um processo de desmoralização progressiva dos direitos sociais dos trabalhadores, articulado como sinalização às elites econômicas do país. Naquele ano, o candidato à vice-presidência da chapa, o militar da reserva Antônio Hamilton Martins Mourão, acusou o 13° salário de ser algo exótico como uma "jabuticaba" (Seto, 27 set. 2018; Galvani, 30 set. 2019). Mais adiante, já no exercício do mandato, Bolsonaro anunciou o pagamento de uma 13ª parcela aos beneficiários do PBF (Seto, 27 set. 2018), projeto que não se efetivou. Essas e outras idas e vindas de propostas para modificações no PBF e de ameaças a direitos consolidados confluíram no objetivo de progressivamente desmoralizar o programa e a estrutura da rede de proteção social.

É importante sublinhar que o PBF foi um programa que sintetizou mudanças na forma de conceber as políticas sociais no país. Ao conciliar os interesses de mercado com os de parcela da população empobrecida, viabilizou a coesão social em torno da ideia de transferência condicionada de renda no país. Sua estrutura, ordenada em torno das condicionalidades (De Sordi, Gonçalves-Neto, 2021), permitiu a construção de certa compreensão em torno das condições de vida de pessoas consideradas pobres a partir de critérios fincados em linhas de corte de renda, deixando em subterrâneo (De Sordi, 2019, p.279) o conservadorismo moral (Bello, 2016) que dominava esse campo e que começou a se manifestar de forma generalizada com a campanha presidencial de Bolsonaro em 2018 que, por sua vez, se utilizou das acusações feitas pelo governo Temer de desvios de verbas no PBF (Fernandes, 8 out. 2018). Estas serviram, ainda em 2016, de impulso para o encolhimento inicial do programa (Campello, Brandão, 2 jun. 2022), sob o argumento de necessidade de "pente-fino" nos programas sociais para melhoria do instrumento de focalização.

Como toda política que corresponda à agenda neoliberal, a desmoralização do Estado (Harvey, 2014) abre caminho para um tipo de remoralização conservadora que engendra mecanismos de convencimento sobre a necessidade de mudanças em estruturas já existentes, em um processo de contrarreforma que tem como alvo, especialmente, as políticas ligadas aos direitos sociais (Behring, 2003). Desfazer a rede de proteção social e finalizar o PBF demandou a publicização não só de supostos problemas nos programas sociais, mas o "teste" da imagem pública das propostas de programas que poderiam ser erguidos em seu lugar.

Assim, ao longo de 2020, o governo federal fez circular publicamente a informação de que buscava um substituto para o PBF. A proposta denominada Renda Brasil, apesar de nunca formalmente apresentada (Novaes, 15 set. 2020), previa a redundância de unificar ações socioassistenciais e tencionava gerar uma "marca social" para o governo. O Renda Brasil foi interditado em meados de setembro daquele ano por sua impopularidade ao sinalizar que alteraria programas como o Benefício de Prestação Continuada (BPC). Os atritos de Bolsonaro com seu vice em relação ao programa foram levados a público como desentendimento de arbitragem no governo, e o desfecho da proposta foi explicitado na fala de Bolsonaro: "No meu governo, até 2022, está proibido falar a palavra Renda Brasil. Vamos continuar com o Bolsa Família e ponto final" (Uribe, Caram, Pupo, 15 set. 2020).

Por ser um programa socialmente reconhecido e politicamente legitimado, a desmoralização do PBF e, principalmente, de seus beneficiários foi essencial para que pudessem ser propostas alterações em sua estrutura. Diante da impopularidade das alterações até então sugeridas, a suposta necessidade de medidas urgentes e extraordinárias para alterações no campo social ganhou fôlego em meio à emergência social gerada pela pandemia de covid-19. Progressivamente, com a implementação do Auxílio Emergencial, o foco do debate público recaiu sobre os valores a serem transferidos à população, em desfavor da rede de serviços socioassistenciais, ao passo que o papel do PBF, desarticulado pela descontinuidade do uso do CadÚnico, do Cras e do Suas, passou ao de um programa acessório, supostamente insuficiente para o contexto pandêmico.

Elaborado por uma frente política diversa, o Auxílio Emergencial, criado pela lei n.13.982, de 2 de abril de 2020, entrou em funcionamento a despeito da existência dos instrumentos de gestão do PBF (De Sordi, 24 ago. 2021) e dos demais programas e instrumentos de gestão social – apenas incorporando automaticamente os beneficiários desse programa ao Auxílio Emergencial. Antes de ser implementada, a proposta do Auxílio Emergencial passou por uma discussão pública que, em geral, confundiu as expressões renda básica e renda mínima, e, por fim, foi fincada na definição dos valores a serem pagos à população.

O Auxílio Emergencial, por tais características, foi um programa elaborado sem discussão ampla com o Suas e seus órgãos e sem a utilização do CadÚnico (Mello, 25 jan. 2021; De Sordi, 2 fev. 2021), priorizando o cadastramento para o recebimento do auxílio por meio de um aplicativo de celular (Branco, 7 abr. 2020) lançado pela Caixa Econômica Federal, responsável por gerir, entre outros, os recursos e as transferências de dinheiro para os programas sociais do governo federal. Ao dissociar a transferência de dinheiro das ações socioassistenciais, a implementação do Auxílio Emergencial não só gerou distorções e dificuldades para os sujeitos se cadastrarem (Lacerda, 13 nov. 2021, 2022), como contribuiu para a aceleração do sucateamento da rede dos Cras (Schiaffarino, 1 jan. 2022), com a consequente formação de filas em frente às agências da Caixa Econômica Federal (Baptista, 7 maio 2020), mas provocou a mobilização pelo apagão dos dados sobre desigualdade social no país. A não utilização do CadÚnico como forma prioritária de cadastramento para o acesso ao Auxílio Emergencial foi uma espécie de experimentação para as alterações de gestão dos programas sociais que provocariam o desmanche da rede de proteção social, o que se confirma em 2021 com o fim do PBF e o lançamento do PAB.

Esse é um ponto que permite evidenciar que, na dimensão de programas sociais organizados por postulados neoliberais (Mauriel, 2008), as bases informacionais são cruciais para o tipo de decisão que será tomada para orientar e conservar a execução das políticas sociais. Como nota Sen (2010, p.78-83), a base informacional que é utilizada na tomada de decisões de gestão para políticas sociais permite gerar um juízo que é também um produto social, e, nesse aspecto, é preciso considerar que as informações que são excluídas, apesar de não influenciarem diretamente os juízos de avaliações técnicas e de gestão que serão feitos com os dados disponíveis, estão subjacentes a eles.

Ao eliminar a etapa de utilização do CadÚnico em seus aspectos de mapeamento de perfil social da população atendida, o governo federal atribuiu o tom de sua própria interpretação para o crescimento da pobreza, relacionando-a ao contexto de crise econômica decorrente da pandemia de covid-19 no país, ou seja, enquanto algo conjuntural (Borges, 19 maio 2022; Não culpe..., 30 abr. 2021). Os dados exigidos para o cadastro e recebimento do Auxílio Emergencial por meio do aplicativo da Caixa foram definidos em torno da renda e do emprego (Brasil, 3 fev. 2022), desconsiderando outros de maior complexidade que eram coletados com o CadÚnico (De Sordi, 2019) e que permitiam compreender fatores estruturais relacionados à reprodução da condição de pobreza.

O interesse do governo federal em eliminar dados de maior complexidade, maquiando assim o progressivo e acelerado empobrecimento populacional, informa certo rearranjar do perfil que define os parâmetros de leitura da estratificação social do país e que tem sido observado pelas ciências humanas e sociais, de forma ainda difusa, nos últimos anos; de que o perfil dos sujeitos em condição acelerada de empobrecimento tem se alterado em um efeito cascata derivado das reformas macroeconômicas (Fiocruz, 5 dez. 2018) levadas a cabo no governo Temer e aprofundadas no governo Bolsonaro.

Esse processo, ainda recente, implica a necessidade de redefinição do próprio entendimento socialmente construído em torno da condição de “pobreza” (Telles, 2013) como chave de leitura para a ampliação do perfil dos trabalhadores atendidos por programas sociais e para as modificações encaminhadas pelo PAB na oferta de serviços socioassistenciais. Com o PBF desarticulado e finalizado, o Auxílio Emergencial e o PAB cumpriram o papel de incorporar parcelas populacionais que passaram a ser identificadas como “pobres”, porém, não nos patamares morais estabelecidos anteriormente aos beneficiários do PBF (De Sordi, 2019), estes últimos constantemente culpabilizados por sua pobreza sob a acusação de formas e modos de vida que levariam a essa condição por inação pessoal; argumento utilizado publicamente mais de uma vez por Bolsonaro (Mendes, 10 ago. 2021; Bolsonaro..., 28 out. 2021) e formalizado na regulamentação do Auxílio Brasil com o objetivo do programa definido pelo “estímulo ao esforço individual” (De Sordi, 24 ago. 2021).

Em meio à conjuntura de empobrecimento, mortes por covid-19 e o retorno do desemprego e da fome ao país, o Auxílio Emergencial cumpriu um papel urgente e necessário; em seu auge, atendeu a 67,9 milhões de trabalhadores, garantindo patamares mínimos de subsistência (Beghin, 28 fev. 2021) e dinheiro aos trabalhadores informais, desempregados e, portanto, fora dos circuitos de atendimento socioassistencial. Sua abrangência e popularidade, porém, confluíram para a definição de uma marca para o governo e a alteração dos valores sociais de leitura da condição de pobreza. Mais que uma mudança técnica de um programa para outro, temos não só o encerramento do PBF, mas o encerramento da coesão social em torno das formas para o combate à pobreza e à extrema pobreza no país. Não se tratou de mera substituição de um programa por outro.

O PAB foi instituído com o argumento da urgência de lidar com o “contexto do final do Auxílio Emergencial e os desafios da situação de pós-pandemia” (Brasil, 5 ago. 2021) em um processo de implementação realizado sem discussão com as estruturas do Suas, ou o envolvimento da sociedade. A gestão do programa se articulou às práticas clientelistas e de filantropização da pobreza (Paoli, 2002), em uma linguagem semelhante, porém diferente, daquela dos anos 1990, de modo que o Auxílio Brasil reativou, por exemplo, debates em torno de *vouchers* ligados ao acesso à educação e à alimentação.

Como exposto na lei n.14.284 (Brasil, 29 dez. 2021), que institui o PAB, está em cena o retrocesso da "doação direta e 'indireta' de renda" (Art. 2°, inciso I; destaque meu), prática amplamente combatida por seu potencial de fragilização dos objetivos dos serviços socioassistenciais (De Sordi, 2019) a partir do estabelecimento da Lei Orgânica de Assistência Social, do Plano Nacional de Assistência Social e dos instrumentos de gestão do Suas. A diretriz que prevê o "fortalecimento das ações do Sistema Único de Assistência Social (Suas)" (Art. 1°, inciso I), uma proposta vazia diante do sucateamento dos Cras e o desinvestimento no Suas, que, ao longo de 2021, teve seu orçamento reduzido em 60%, sendo que, "desde o início da gestão Bolsonaro, houve uma redução de mais de 70% nos repasses ao sistema, que caiu de R$3 bilhões, em 2019, para R$910 milhões no ano passado" (Schiaffarino, 1 jan. 2022).

O programa prevê, como seus fundamentos e principais ações, o "incentivo ao 'esforço individual'", "a inclusão produtiva rural e urbana, com vistas à 'emancipação cidadã'" (Art. 2°, inciso V; destaques meus), e em seu inciso VII, ações para "estimular a emancipação das famílias em situação de pobreza e de extrema pobreza, principalmente, por meio", entre outros, do "incentivo ao 'empreendedorismo, ao microcrédito e à inserção no mercado de trabalho formal'" (§ 1°, inciso VII, item c; destaques meus), ao passo que das diretrizes do programa é possível destacar, dentre outros: "a 'educação e a inclusão financeiras' das famílias beneficiárias" (§ 2°, inciso IX; destaques meus).

Com o PAB, a pobreza passa institucionalmente a ser lida, isto é, orientando as ações socioassistenciais promovidas pelo Estado, como ausência de esforço individual, ignorância ou displicência no manejo de parcos orçamentos domésticos, como problemas passíveis de ser solucionados com a pedagogia (Cunha, 2018) da promoção de "educação financeira". Cabe destacar que o programa se organiza a partir da desresponsabilização do Estado pela produção e reprodução da pobreza, por meio da culpabilização dos sujeitos empobrecidos por sua própria condição material de vida. Ao centralizar diretrizes e ações nos indivíduos, há a formalização de pressupostos que moralizam a pobreza indicando a promoção da ideia de suposta possibilidade de "esforço individual" no âmbito da individualização de questões que são estruturantes da formação social brasileira, reduzindo o enfrentamento de problemas estruturais aos sujeitos e ao núcleo familiar.

Deve-se considerar nesses pressupostos a parcela de expressão dos valores morais criados pelo PBF com o objetivo de manter a coesão social em torno do programa. Esses não se desfizeram da noção de esforço individual por completo, que esteve sempre presente nas condicionalidades do programa, por sua vez, carregadas do deslocamento da noção de "trabalho" para o papel de referência à moralização dos "pobres" e da "pobreza" (De Sordi, 2019). Ou seja, mesmo quando o emprego formal não é um horizonte possível para todos os sujeitos, a referência à postura do esforço individual do trabalhador permaneceu subjetivada na gestão social dos programas sociais de minoração da pobreza, na formulação da própria imagem do "pobre" a ser atendido pelo PBF (De Sordi, 2021). Imagem essa que agora precisa ser reposicionada em relação ao cenário de interdição da proteção de patamares mínimos pelo Estado a uma parcela ampliada de trabalhadores.

O cenário atual, derivado dessas ações e pressupostos, pode ser capturado pelos dados divulgados no relatório "Insegurança Alimentar e Covid-19 no Brasil", publicado pela rede

Penssan (2022, s.p.) em articulação com ONGs nacionais e internacionais: “enquanto em 2020 a fome estava presente em 3,3% dos domicílios onde a pessoa responsável tinha emprego formal, em 2021/2022 o percentual subiu para 7,3%. Nos domicílios em que o trabalho da pessoa de referência é informal, o aumento foi de 14,3% para 20,3%”.

Há, portanto, uma parcela expressiva de trabalhadores que passou por um processo acelerado de empobrecimento e que agora se vê nos degraus de privação material da população que já era atendida pelos programas sociais, por estar nas linhas de corte de renda definidas como de “pobreza” e de “extrema pobreza”. O aumento dos números do emprego desregulamentado, informal e precário se traduz também nos números de atendimento do Auxílio Emergencial, para o qual, das “96 milhões de pessoas [que] acessaram o aplicativo do auxílio emergencial de R$600. ... 50,5 milhões foram considerados elegíveis para receber o benefício” (Baptista, 7 maio 2020).

No início de 2020, 46 milhões de brasileiros, informais, autônomos e desempregados, que não constavam no CadÚnico, precisaram do Auxílio Emergencial para sobreviver (Auxílio Emergencial..., 26 abr. 2020). Com o fim do Auxílio Emergencial, 38 milhões de trabalhadores não cadastrados anteriormente no CadÚnico não seriam atendidos pelo Auxílio Brasil (Invisíveis..., 6 out. 2020). A desconexão entre os direitos sociais e o trabalho, acentuada pela reforma trabalhista (Braga, 1 maio 2018), já mostrava também seus efeitos e a precarização dos vínculos empregatícios, configurando o desemprego de 14,7% da população e salários insuficientes a 72,4% das famílias de trabalhadores, de acordo com dados da Pesquisa de Orçamentos Familiares (POF) de 2021 (POF 2017-2018..., 19 ago. 2021).

Esses sujeitos têm sido chamados de “invisíveis” (Marchesan et al., 22 nov. 2020) pelo governo federal, sob o argumento de sua não inserção orgânica ao sistema bancário do país. Entretanto, são aqueles que compõem o precariado; estão na base do próprio modo de produção capitalista, como integrantes da classe trabalhadora e na expressão mais crítica do processo de mercantilização do trabalho (Braga, 2012).

Entrementes, a publicização desses números em meio à pandemia revelou não só o trato do governo federal às condições materiais de vida da população em relação ao grau de pauperização generalizada: “Nós encontramos mais de 21 milhões de invisíveis, é muito maior do que a gente imaginava”, mas também revelou ao governo um contingente potencial de trabalhadores empobrecidos, no interior dos programas sociais, com potencial de financeirização diferente daquele atingido pelo PBF:

> Há uma externalidade muito importante nesse processo que é o fato de que nós estamos ‘bancalizando’. Vamos chegar próximo a 25 milhões de pessoas com contas digitais, pessoas que eram completamente desconhecidas do Estado. Hoje a gente tem a conta dessa pessoa, a sua composição familiar, a sua residência, o seu CPF. Quer dizer, são pessoas, passando esse episódio da pandemia, para as quais nós vamos desenvolver aqui – e vamos fazer isso junto com o Parlamento brasileiro – programas de estímulo ao empreendedorismo, aperfeiçoamento, microcrédito; poder chegar perto dessas pessoas para que elas possam se desenvolver (Baptista, 7 maio 2020).

A intenção de “bancalizar” os sujeitos se revelará, com o PAB, em um campo de intervenção, abertura e gestão de processos de financeirização (Lavinas, Gentil, 2018) acentuada do acesso aos direitos sociais, pela liberação do acesso ao crédito consignado e pela distorção do papel social dos programas sociais (De Sordi, 24 fev. 2022) que garantiam, ainda que de forma focalizada tal como era feito pelo PBF, o controle dos níveis de reprodução da pobreza no país. Retrocessos sociais como o desse período recente acabam por colocar em xeque o próprio alcance histórico de medidas como o PBF, do objetivo do programa de quebra intergeracional de pobreza e a potencialidade que apresenta, ou não, para intervir nas questões sociais estruturantes da sociedade brasileira. Não se pode esquecer que o debate no início dos anos 2000 também reposicionou a "pobreza" que,

> por séculos [foi] vista como empecilho à modernidade e ao desenvolvimento do país, a pobreza foi abertamente tida, nos anos iniciais da década de 1990, como dialeticamente oposta às perspectivas abertas pelo fim do militarismo (1985), pela Constituinte (1988) e pelo Brasil pós-Collor (1993). Entretanto, deparamo-nos ... com uma comemoração singular ... [na qual] a pobreza faz-se agora aliada do modelo (desmemoriado e hegemônico) de capitalismo que vivemos (Morais, 2013, p.292).

Com o PAB, a pobreza volta a ser reinterpretada. Passa à condição de mero efeito da crise pandêmica e da inação dos indivíduos, demarcando uma virada de orientação conservadora no histórico dos programas sociais da Nova República, justamente porque alterou o pacto social anterior, expresso principalmente na conciliação de antagonismos sociais operada pelo PBF; programa que era, até então, a síntese de um processo de reformas nos programas sociais.

## Considerações finais

Acentuadamente em 2020 e 2021, panelas vazias e ossos de boi passaram a figurar no espaço público brasileiro como símbolos de denúncia e de protesto, em uma gramática social que lê a queda de condições materiais de vida caracterizada pela formação de "garimpos dos ossos" (De Sordi, 19 out. 2021) e circuitos de distribuição de alimentos, seja por organizações não governamentais (ONGs), ou por meio da abertura de cozinhas solidárias por movimentos sociais, especialmente, pelo Movimento dos Trabalhadores Sem Teto (MTST, 2022). Porém, tais mobilizações têm ocorrido em sentido diverso daquelas dos anos 1990 que disputaram um projeto de sociedade que influenciou o desenho do Fome Zero e do PBF, em um contexto reorganizado pela "hegemonia às avessas" (Oliveira, 2010) dos anos 2000.

Em julho de 2021, circularam nos jornais notícias sobre a formação de filas de famílias de trabalhadores na porta de açougues para recolher doações de restos de ossos de animais que seriam descartados. Uma das mulheres que recolhia os restos em um caminhão na cidade do Rio de Janeiro afirmou "ou comemos isso, ou morreremos de fome", outra contou ao jornalista que registrou a recolha que há seis meses vai até o caminhão com os ossos: "pego aqui há seis meses ... . É uma ajuda e tanto! Pego, levo e salgo" (De Sordi, 19 out. 2021). Em agosto, o preço da cesta básica atingiu um salário-mínimo nas principais capitais do país (Chiara, 13 ago. 2021).

Em setembro do mesmo ano, no dia 23, o MTST ocupou a sede da Bolsa de Valores, em São Paulo. Durante o protesto, manifestantes empunharam ossos de boi em frente ao painel luminoso que exibe os indicadores econômicos. Cartazes erguidos durante o protesto denunciavam que "tem gente ficando mais rica com a nossa fome". A bandeira nacional teve o emblema positivista de "Ordem e Progresso" substituído pela palavra "fome" e foi pendurada nas paredes em protesto (De Sordi, 19 out. 2021).

Em 13 de novembro de 2021, o MTST e a articulação Frente Povo Sem Medo reuniram cerca de 20 mil pessoas para a Marcha contra a Fome que percorreu a cidade de São Paulo (Lacerda, 13 nov. 2021). A manifestação também ocorreu na orla do Leblon, um dos bairros mais elitizados da Zona Sul do Rio de Janeiro, e foi realizada por manifestantes do Movimento dos Pequenos Agricultores (MPA) que carregaram a bandeira do movimento e cartazes com mensagens que denunciaram a associação entre concentração de renda e a fome (MPA, 13 nov. 2021). No dia 24 do mesmo mês, moradores da favela de Heliópolis, em São Paulo, organizaram a "Marcha da Panela Vazia", que foi repetida em 21 de dezembro (Novo protesto..., 21 dez. 2021).

Em outubro de 2021, um sujeito em situação de rua que utiliza pedaços de madeira descartada pelos comerciantes da Ceagesp para cozinhar – prática cada vez mais comum, alternada com o uso de álcool, por causa do aumento do preço do gás de cozinha – teve seu dia acompanhado por um jornalista (Santiago, 16 jan. 2021) que relatou:

> 'É o que eu tenho pra hoje', diz Daniel Pereira, 64, após derrubar no chão um filé de peixe empanado e pegá-lo com as mãos. Ele joga um pedaço de madeira no fogo para preparar seu almoço e desvia da labareda que sobe do fogão improvisado com lata de tinta, grade e pedras encontradas na rua. Pereira ergueu um barraco com sobras de madeira em uma calçada próxima ao portão de entrada de pescados da Ceagesp (Companhia de Entrepostos e Armazéns Gerais de São Paulo), maior entreposto de alimentos da América Latina, localizado na Vila Leopoldina, zona oeste de São Paulo.

Ao longo de 2021 e 2022, trabalhadores organizados na Federação Única dos Petroleiros (FUP) desenvolveram ações de distribuição de botijões de gás nas periferias das cidades do país, em mobilizações pela denúncia da política de preços adotada pelo governo federal e da carestia (Petroleiros..., 1 fev. 2021).

Essas manifestações, observadas em conjunto, instituem uma gramática simbólica de protestos públicos e permitem evidenciar a percepção de um novo tipo de dinâmica do social (Williams, 2013, p.149) construída a partir da experiência de degradação acelerada das condições materiais de vida dos trabalhadores. Há um processo de reinvenção e de ressignificação da pobreza que lê esse mesmo processo nos termos de certa institucionalidade expressa nos programas sociais, porém, como forma de organização das relações sociais que têm se expressado a partir do acúmulo da experiência social de desenvolvimento e de redução dos níveis de desigualdade entre 2003 e 2015. Há um antes e um depois que organizam essas experiências de mobilização.

A inscrição dessa gramática social de protestos e reivindicações traduzida na cena pública a partir da exibição de ossos de animais, panelas vazias e distribuição de gás dialoga e interpreta os limites de uma conjuntura de massacre dos trabalhadores e busca antagonizar com a proposta do ideal meritocrático do indivíduo mobilizado pelo trabalho como referencial de valor e da moralização da pobreza. Os trabalhadores, por sua vez, movem o moinho das reformas neoliberais traduzidas na necessidade de ajustes macroeconômicos frente ao "custo" social, ao passo que "pobreza" se reconfigura progressivamente em condição de "fome" para separar os "pobres" dos "não pobres", independentemente de seu *status* de emprego, isto é, de classe, que agora precisa ser varrido da discussão. Assim, talvez esteja em pauta uma diferente leitura da "pobreza", que busca integrar aos circuitos de financeirização os trabalhadores empobrecidos em anos recentes e não acostumados à nova titulação.

Em vídeo recente de campanha eleitoral, Bolsonaro apresenta o seu estandarte de campanha: o Auxílio Brasil como programa melhor que o PBF (e como alternativa a Lula, também candidato), justamente porque incentivaria o trabalho, já que, em uma distorção dos critérios de elegibilidade do PBF, anuncia que os beneficiários não perderão o direito ao recebimento caso "arrumem emprego". Sentados em cadeiras de plástico dispostas em um círculo junto a mulheres que figuram no papel de beneficiárias do programa, uma delas diz "a minha irmã perdeu [o PBF] porque comprou uma geladeira", ao que Bolsonaro comenta: "era para te manter escravizada. Se tiver emprego agora, não perde o Auxílio Brasil" (Santiago, 20 jun. 2022).

Estão mobilizadas duas estruturas de ideias (Williams, 2013) que têm aberto caminho para o desmanche da rede de proteção social: a liberdade individual propagada como valor fundante da economia liberal e o acesso ao consumo atrelado a esta liberdade, sem intermédio do Estado ou de qualquer tipo de associação que não aquela de ordem doméstica. Trata-se não mais da existência de alguma coesão social que promova a democracia; o espaço público para a formulação e o pensar a política. O PAB representou o fim do PBF, mas também das referências públicas que conformavam noções de cidadania anteriormente constituídas.

AGRADECIMENTOS

Este artigo apresenta os resultados parciais das pesquisas de pós-doutoramento "Programas sociais brasileiros: Pobreza, fome e desenvolvimento social (2016-2021)", desenvolvida com o apoio financeiro da Casa de Oswaldo Cruz/Fiocruz, e "As reformas nos programas sociais no Brasil e a desativação do espaço público: solidariedade social, pobreza e mobilizações", desenvolvida no Departamento de Sociologia da FFLCH/USP, com vinculação ao Centro de Estudos dos Direitos da Cidadania.

## NOTAS

[1] O PBF foi criado em 2003 pela medida provisória (MP) n.132, convertida na lei n.10.836, de 9 de janeiro de 2004, e regulamentado pelo decreto n.5.209, de 17 de setembro de 2004. Foi finalizado pela MP n.1.061, de 9 de agosto de 2021, que instituiu o Auxílio Brasil e modificou o PAA, convertido na lei n.14.284, de 29 de dezembro de 2021, alterada pelo decreto n.10.870, de 25 de novembro de 2021, e pela MP n.1.106, de 17 de março de 2022, ampliando a financeirização por meio do crédito consignado.

[2] São exemplos, o Programa de Cisternas, Luz para Todos, programas voltados para a soberania e segurança alimentar e nutricional, reforma agrária, entre outros.

[3] A ação Brasil Carinhoso, de 2012, tinha por objetivo aprofundar a estratégia Brasil sem Miséria, de 2011. Ambas se complementavam com ações entre diferentes ministérios, com ações de busca ativa de beneficiários para os programas sociais, ampliação da faixa de atendidos, medidas de incentivo à educação, saúde e alimentação adequada na primeira infância.

[4] Como o PBF, o BPC, a Tarifa Social de Energia Elétrica, Programa de Cisternas, Isenção de Pagamento de Taxas de Inscrição em Concursos Públicos, Programa Nacional de Fortalecimento da Agricultura Familiar (Pronaf), Programa de Erradicação do Trabalho Infantil (Peti).

[5] O IGD foi criado em 2006, para medir a qualidade da gestão do CadÚnico e acompanhar o cumprimento de condicionalidades dos programas no nível municipal, transferindo recursos suplementares para os municípios, incentivando a melhoria da oferta de ações sociais.

[6] Medida provisória n.870, de 1 de janeiro de 2019, provocou alterações no Sistema Nacional de Segurança Alimentar e Nutricional (Sisan) e a extinção do Consea, com o efeito cascata em seus órgãos em nível estadual e municipal. A MP foi convertida na lei n.13.844, de 2019.
